# The Non-Panel Movement

**Published:** 1913-11-01

**Authors:** 


### THE NON-PANEL MOVEMENT.

### Some Telling Facts on the Panel System.

Last week the correspondence columns of The Hospital contained a letter signed by the pseudonym of "Northerner," which not only revealed some interesting facts concerning panel practitioners and panel practice in one of the large cities of the North of England, but also contained the writer's personal views and suggestions for a solution of the panel problem as it presents itself in his locality and in a good many others of a similar urban character. The expedient he advocated was distinctly novel and daring, for it was nothing less than compulsory panel practice for all doctors on the active list. Some obvious objections to this drastic proposal are voiced in the letter printed below, which we have received from a practitioner in the South of England signing himself "Southerner." The arguments he advances are certainly formidable obstacles to the proposals of "Northerner," and are probably insuperable in a country having the traditions and tendencies of extreme personal liberty which prevail in Great Britain.

As time goes on and experience of the actual working of the panel system accumulates, its many weak places are becoming more and more evident. The *Lancet*, for instance, publishes regularly the reports of a special commissioner who is investigating every aspect of medical practice under the Insurance Act in different parts of the Kingdom. Nottingham has lately been visited, and, as usual, some facts emerge which are curious commentaries upon the rhodomontade of the political upholders of the Act and everything connected with it. One practitioner, he learned, actually saw one hundred panel patients in one evening, and "went to bed exhausted and heartily ashamed of himself"; we are not told, however, that his repentance lasted into the next morning sufficiently to induce him to remove his name from the panel. The panel doctor, it is only too evident, who attempts to diagnose and treat patients in this wholesale fashion, must be nothing else but an automatic distributor of pills and potions. The "latest aids to diagnosis," the efficient service by first-class doctors, and all the rest of the purple rhetoric of the days when the Act was still a Bill, look rather improbable from panel men such as this. And the *Lancet's* commissioner also discovered that the evil of over-swollen panel lists is not serious in Nottingham; very few of the panel practitioners have accepted over 2,000 names. If incidents such as this of the hundred patients per evening happen in practices of 2,000 insured, what must the evening surgery be like when there are 6,000 or 7,000 names on the list? "More thrue than tellable," as Privit Mulvaney used to say, we shrewdly suspect.

A painful incident in one of the Eastern Counties casts yet another lurid light on the bluffing of the profession last January by the threat of imported whole-time panel doctors. At one of the very few towns where this actually happened, the practitioner concerned was one whose record was distinctly bad; he had actually been summarily dismissed from a resident appointment at a well-known London hospital, an incident so rare as in itself to stamp him as undesirable. Lately a charge of criminal libel was brought against him, and early this week he was found dead in circumstances strongly suggestive of suicide. We should not dwell on this sad wreck of a career did it not illustrate so completely the means employed to fill the panels at any cost and anyhow in January, and the absolute disregard for the kind of service supplied to the insured so long as the medical profession could be cowed and intimidated into accepting the terms offered.

